# Supplementary material for: Bidirectional Regulatory Effects of Warming and Winter Snow Changes on Litter Decomposition in Desert Ecosystems
Source: Plants (Basel). 2025 Sep 2;14(17):2741. doi: 10.3390/plants14172741 (PMC12430361; doi:10.3390/plants14172741)
Supplement: Supplementary file 1 [file plants-14-02741-s001.zip › plants-3756442-supplementary.pdf]

Table S1. Results of two-way ANOVA on the effects of warming (T), winter snow changes (S), and their interactions on nitrate nitrogen (NO_3^--N), ammonia nitrogen (NH_4^+-N) organic carbon (SOC), pH, electrical conductivity (EC). *p* values in bold are significantly different ($p < 0.05$).

	Treatments	NO_3^--N ($g\ kg^{-1}$)	NH_4^+-N ($g\ kg^{-1}$)	SOC ($g\ kg^{-1}$)	pH	EC
March	S	**P<0.05**	0.085	0.361	0.883	**P<0.01**
	T	0.381	0.103	0.469	0.23	0.319
	S*T	0.333	0.376	0.989	0.808	**P<0.05**
May	S	**P<0.001**	0.533	**p<0.001**	0.74	**P<0.01**
	T	0.488	0.553	**p<0.01**	0.533	0.603
	S*T	**P<0.01**	0.5	**p<0.001**	0.471	0.791

Table S1. Impact of warming and winter snow changes on soil physicochemical properties (i.e., EC, pH, SOC, NH_4^+-N, and NO_3^--N) in original data after snow melt (a, March) and at the end of growing season (b, May), respectively. +T, warming; CK, ambient temperature; +S, 100% snow addition; CK, ambient winter snow; -S, 100% snow removal. EC, electrical conductivity; SOC, soil organic carbon; NH_4^+-N, ammonium nitrogen; NO_3^--N, nitrate nitrogen.

	Treatments	NO_3^--N (g kg^{-1})	NH_4^+-N (g kg^{-1})	SOC (g kg^{-1})	pH	EC
March	+S+T	2.65±0.08	2.86±0.22	3.27±0.56	8.5±0.27	169.43±5.2
	+S+CK	2.58±0.13	2.81±0.34	3.07±0.3	8.75±0.33	169.64±2.89
	CK+T	3.29±0.77	3.12±0.92	3.35±0.46	8.58±0.34	174.86±2.63
	CK+CK	2.59±0.07	4±1.08	3.23±0.54	8.63±0.33	170.6±0.85
	-S+T	3.47±0.8	2.7±0.46	3.63±0.87	8.57±0.33	174.3±7.72
	-S+CK	3.6±0.83	3.24±0.47	3.47±0.16	8.81±0.46	185.13±8.69
May	+S+T	2.71±0.56	2.94±0.45	3.7±0.98	7.37±0.04	259±6.03
	+S+CK	2.76±0.48	3.23±0.33	2.21±0.97	7.46±0.03	261±7.79
	CK+T	3.95±0.62	2.84±0.37	4.03±1.25	7.51±0.28	246.4±36.52
	CK+CK	3.05±0.12	2.96±0.17	3.87±1.29	7.42±0.04	241.33±17.98
	-S+T	4.52±0.81	3.14±0.09	3.79±0.83	7.57±0.28	226.52±21.3
	-S+CK	5.89±0.71	3±0.54	4.11±1.16	7.41±0.27	203.63±20.59

Table S2. Results of two-way ANOVA on the effects of warming (T), winter snow changes (S), and their interactions on soil enzyme activities after snow melt (March) and at the end of growing season (May), respectively. AG, α-glucosidase; BG, β-glucosidase activity; NAG, β-1,4acetamido-glucosidase; ALP, alkaline phosphatase. *p* values in bold are significantly different ($p < 0.05$).

Time	Treat-	AG (nmol $g^{1}h^{-1}$)	BG (nmol $g^{1}h^{-1}$)	Urease (nmol $g^{1}h^{-1}$)	NAG (nmol $g^{1}h^{-1}$)	ALP (nmol $g^{1}h^{-}$
March	S	**P<0.001**	**P<0.001**	0.743	**P<0.001**	**P<0.001**
	T	**P<0.001**	**P<0.001**	**P<0.001**	**P<0.001**	**P<0.001**
	S*T	**P<0.001**	**P<0.001**	**P<0.001**	**P<0.001**	0.137
May	S	**P<0.001**	**P<0.001**	**P<0.001**	**P<0.001**	**P<0.001**
	T	0.281	0.607	0.051	**P<0.001**	**P<0.001**
	S*T	**P<0.001**	**P<0.001**	**P<0.001**	**P<0.001**	**P<0.001**

Table S3. Results of three-way ANOVA on the effects of warming (T), winter snow changes (S), time, and their interactions on the decomposition rate (k) of red tea, green tea and *Erodium oxyrhinchum* at different locations, respectively. *p* values in bold are significantly different ($p < 0.05$).

Location	Treatment	Red tea K (g $g^{-1}m^{-1}$)	Green tea K (g $g^{-1}m^{-1}$)	*Erodium oxyrhinchum* K (g $g^{-1}m^{-1}$)
Aboveground	S	**P<0.001**	**P<0.001**	**P<0.001**
	T	**P<0.001**	**P<0.001**	**P<0.001**
	Time	**P<0.001**	**P<0.001**	**P<0.001**
	S*T	0.828	**0.05**	**P<0.001**
	S*Time	**P<0.05**	**P<0.001**	**P<0.001**
	T*Time	**P<0.001**	**P<0.001**	**P<0.001**
	S*T*Time	0.83	**P<0.001**	**P<0.001**
Belowground	S	**P<0.001**	**P<0.001**	**P<0.001**
	T	**P<0.001**	**P<0.001**	**P<0.001**
	Time	**P<0.001**	**P<0.001**	**P<0.001**
	S*T	0.663	0.097	**P<0.001**
	S*Time	**P<0.001**	**P<0.001**	**P<0.001**
	T*Time	**P<0.05**	0.139	**P<0.001**
	S*T*Time	0.896	**P<0.01**	**P<0.001**

Table S4. Values of decomposition rate (k) and stabilization coefficient (S) with standard deviation for different sites, corresponding to the legend of Figure 5.

Site	S	K
CN Semi-arid desert sandy	0.31±0.1	0.0104±0.0039
CN Semi-arid desert loamy	0.54±0.15	0.0035±0.0034
NL Wet forest	0.13±0.01	0.0134±0.0011
IE Oceanic raised bog disturbed	0.23±0.05	0.0094±0.0009
IE Oceanic raised bog undisturbed	0.2±0.01	0.0102±0.0007
GCE-LTER minerogenic salt marsh(0-10cm)	0.17±0.02	0.0072±0.0007
CN GD	0.71±0.05	0.003±0.0006
IS Geothermal wet grassland warmed	0.04±0.11	0.0187±0.0059
IS Geothermal wet grassland ambient	0.15±0.1	0.0204±0.0097
NL Pasture	0.14±0.02	0.0119±0.0007
NL Floating	0.23±0.02	0.0101±0.0014
PA Lowland tropical forest	0.06±0.09	0.039±*

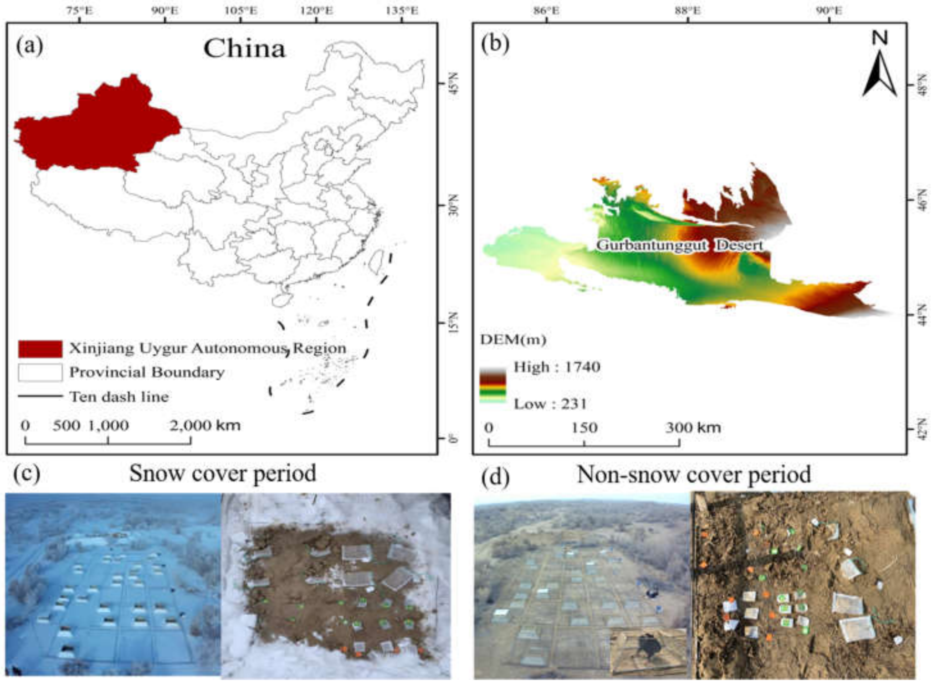

Figure S1. Location map of the study area: geographic location of Xinjiang (a); geographic location of the Gurbantunggut Desert (b); realistic picture of study site and litter bag application during snow cover period (c); realistic picture of study site, open top chamber, and litter bag application during non-snow cover period (d).
